# Proof of Principle: Short-Term Sulforaphane Precursor Intake Alters Selected Circulating Oxidative Stress and Muscle Damage Biomarkers Following Maximal Exercise: A Double-Blind Crossover Trial

**DOI:** 10.3390/nu18162617

**Published:** 2026-08-10

**Authors:** Ruheea Taskin Ruhee, Shiori Wakasugi-Onogi, Sihui Ma, Nobuhiro Nakamura, Yasuhiro Seki, Katsuhiko Suzuki

**Affiliations:** 1 Graduate School of Sport Sciences, Waseda University, Tokorozawa 359-1192, Japan; 2 Faculty of Sport Sciences, Waseda University, Tokorozawa 359-1192, Japan

**Keywords:** sulforaphane, exercise, oxidative stress, antioxidant, muscle damage

## Abstract

Background: Endurance exercise is known to induce the excessive production of reactive oxygen metabolites due to enhanced metabolic activity and inflammation, leading to oxidative stress and related injuries. Sulforaphane (SFN), an isothiocyanate, is broadly acknowledged for its potential antioxidant and anti-inflammatory properties, along with other protective functions. Objective: The purpose of this study was to investigate the effects of short-term SFN precursor supplementation on maximal exercise-induced muscle damage, oxidative stress and endurance capacity in healthy adults. Methods: In a double-blind placebo-controlled crossover design, 14 healthy participants (7 males, 7 females; age, 25.07 ± 1.04 years; weight, 65.28 ± 4.13 kg; height, 170.07 ± 3.46 cm; BMI, 22.48 ± 0.91) received SFN glucosinolate capsules (200 µmol/day) or placebo for 2 weeks during the first trial. Following a three-week washout period, the participants crossed over to the alternate treatment. Maximal exercise tests were performed following each supplementation (SFN or placebo) period. Blood samples were collected at baseline (before treatment), pre-exercise, post-exercise and 2 h after each exercise session. Results: SFN supplementation did not alter endurance capacity or blood cell counts; however, it significantly attenuated serum creatine kinase (CK) activity (*p* = 0.034; SFN, 121.52 ± 16.89; placebo, 291.84 ± 133.07) and the myoglobin (Mb) concentration (*p* = 0.011; SFN, 15.82 ± 1.95; placebo; 27.31 ± 5.71) following exercise. Analysis of oxidative stress markers showed no significant changes in the absolute values; however, in the d-ROMs (*p* = 0.038; SFN, 107.42 ± 2.39; placebo; 112.32 ± 2.11) and OXY-adsorbent tests (*p* = 0.032; SFN, 115.97 ± 4.15; placebo; 107.73 ± 3.88), the percent changes from the baseline showed a significant interaction with the supplement. These findings suggest that short-term SFN supplementation may mitigate exercise-induced muscle damage and oxidative stress, suggesting a modest influence on the circulating biomarkers associated with muscle damage without altering the endurance capacity of healthy people. Conclusion: SFN intake may be a beneficial, non-invasive strategy to modulate acute biochemical responses to exercise via the antioxidant defense system in physically active individuals.

## 1. Introduction

The effectiveness of antioxidant supplementation for enhancing exercise performance remains inconclusive, with studies yielding mixed and sometimes conflicting results. Exercise-induced muscle damage (EIMD) and organ damage are familiar types of inflammation triggered by prolonged or intense exercise. EIMD can be caused by either metabolic or mechanical stress, based on the mode, intensity, duration of exercise, and individual training status [1,2]. Frequent muscle contractions and oxygen metabolism during exercise generate an abundance of reactive oxygen species (ROS) or reactive oxygen metabolites, which can result in oxidative damage to cellular constituents and muscle tissue, decrease muscle contractility, and therefore decrease exercise tolerance [3,4,5]. In this context, antioxidant supplementation may restore redox balance by counteracting excessive ROS production and improving an unfavorable relationship between oxidative reactions and the antioxidant defense system.

Sulforaphane (SFN) is a naturally occurring isothiocyanate, which could potentially reduce exercise-induced oxidative stress and inflammation [6]. SFN is abundantly found in broccoli and broccoli sprouts as glucoraphanin, which is hydrolyzed by myrosinase during cooking and chewing, and converted into bioactive isothiocyanates. Also, SFN indirectly interacts with the major transcription factors Nrf2 (nuclear factor erythroid 2-related factor 2) and nuclear factor kappa B (NF-κB) to reduce oxidative stress and inflammation [7,8,9,10]. Additionally, SFN enhances the working of endogenous antioxidant defense system, thereby attenuating exercise-induced oxidative stress. Recently, Wakasugi et al. reported that SFN attenuated neutrophil-derived ROS production, myeloperoxidase (MPO) degranulation, and phagocytic activity both in vitro and ex vivo, in a dose-dependent manner. These effects were attributed to both the activation of endogenous antioxidant defense systems via Nrf2 signaling pathways and direct antioxidant activity through scavenging hypochlorous acid (HOCl), where SFN more strongly removed HOCl than ascorbic acid (vitamin C) [11]. These findings suggest that SFN may be a promising nutritional strategy for reducing exercise-induced oxidative stress and inflammation.

To evaluate the physiological effects of antioxidant interventions during exercise, reliable biomarkers of muscle damage and oxidative stress are required. Creatine kinase (CK) and myoglobin (Mb) leak from damaged muscle into circulation following intense exercise and have been used as indirect markers of muscle damage [12,13,14]. Derivatives of reactive oxygen metabolites (d-ROMs) and biological antioxidant potential (BAP) tests can be used with small amounts of serum samples. A d-ROMs test is used as a simple clinical marker [15] to quantify systemic oxidative stress, by measuring the hydroperoxides derived from oxidized lipids, proteins, amino acids and nucleic acids [16]. A BAP test analyzes the ability of a compound to neutralize free radicals, thereby providing a complete assessment of oxidative stress [17]. Both d-ROMs and BAP tests have been used to meet most of the key criteria for an ideal oxidative stress biomarker in clinical practice [18,19,20,21], and have also been demonstrated as useful indicators for the assessment of redox status related to exercise-induced changes in blood circulation [22,23,24,25]. In conjunction with d-ROMs and BAP tests, the total serum antioxidant capacity can be assessed by an Oxy-adsorbent (OXY) test, which helps to identify the effectiveness of specific antioxidant treatments by measuring the ability to neutralize HOCl [26]. An OXY test evaluates non-enzymatic antioxidants and their capacity to neutralize ROS [27].

Despite substantial evidence from cellular and animal studies demonstrating the antioxidant and anti-inflammatory effects of SFN, its efficacy for attenuating oxidative stress induced by maximal exercise in healthy humans remains unclear. Moreover, previous exercise-related studies have not comprehensively addressed the combined effects of short-term SFN supplementation on muscle damage, systemic oxidative stress, antioxidant capacity, and exercise performance using an integrated approach with selected biomarkers. Therefore, the present study was designed to determine whether short-term SFN supplementation could attenuate maximal exercise-induced muscle damage and oxidative stress while improving antioxidant capacity and endurance performance in healthy adults. By simultaneously assessing conventional markers of muscle damage (CK and Mb) together with complementary redox biomarkers (d-ROMs, BAP, and OXY), this study provides a comprehensive evaluation of the physiological responses to SFN supplementation during maximal exercise in healthy adults.

## 2. Materials and Methods

### 2.1. Experimental Approach

This study employed a double-blind, placebo-controlled crossover design. Participants completed two intervention periods, each consisting of two weeks of SFN glucosinolate capsule or placebo supplementation, separated by a three-week washout period. Blood samples were collected before supplementation and before, immediately after, and two hours after maximal exercise. The primary outcomes included circulating oxidative stress biomarkers and muscle damage markers. The study protocol was reviewed and approved by the Waseda University ethical committee (2021-220), and conformed to the ethical principles of the Declaration of Helsinki.

### 2.2. Study Subjects

A total of 14 healthy subjects (7 males and 7 females) participated in this study, aged between 21 and 30 years old. All participants were recreationally active (were involved in regular activity and considered fit) and free from chronic illness. Written informed consent was obtained prior to commencement of the study. The participants were examined by a physician using a questionnaire to assess their medical history and any physical conditions that might affect or influence the study findings. Participants with high blood pressure, anemia, asthma, high blood cholesterol or those taking medications or supplements with anti-inflammatory properties were excluded from this study. Female participants were instructed to avoid undergoing the exercise testing during their menstrual period. All the participants were instructed to refrain from drinking alcohol, smoking and doing vigorous exercise on the day prior to the exercise testing.

### 2.3. Intervention

Participants received either SFN glucosinolate capsules (200 μmol/day) or placebo capsules for 2 weeks during the first trial, followed by a three-week washout period, after which they crossed over to the alternate treatment during the second trial. Participants were instructed to take the last capsule 12 h before the test. All the capsules used in this experiment were provided by Murakami Farm Co., Ltd. (Hiroshima, Japan), and were designed to be indistinguishable from each other. The test capsules contained TrueBroc^®^ broccoli seed extract (Brassica Protection Products LLC, Baltimore, MD, USA), a broccoli seed extract standardized to 13% glucoraphanin, together with mustard seed powder as a natural source of myrosinase to facilitate the conversion of glucoraphanin to SFN. A pre-study meeting was conducted, during which the protocol was explained to the participants, and necessary instructions were discussed. The subjects’ body composition was obtained using a bioelectrical impedance analysis (InBody 720, Inbody Japan Inc., Tokyo, Japan). Blood samples were collected before taking the capsules, considering the baseline parameters. Supplement packaging were performed by independent personnel not involved in the data collection to ensure double blinding. The participants were assigned to the initial supplementation sequence using an alternating allocation procedure (capsule A followed by capsule B). The supplements were dispensed in identical containers, and the participants were instructed to maintain their habitual diets for the entire intervention period. The participants recorded their daily capsule intake in a supplementation diary, which was reviewed at the end of each intervention period.

### 2.4. Exercise Protocol

**Figure 1:**
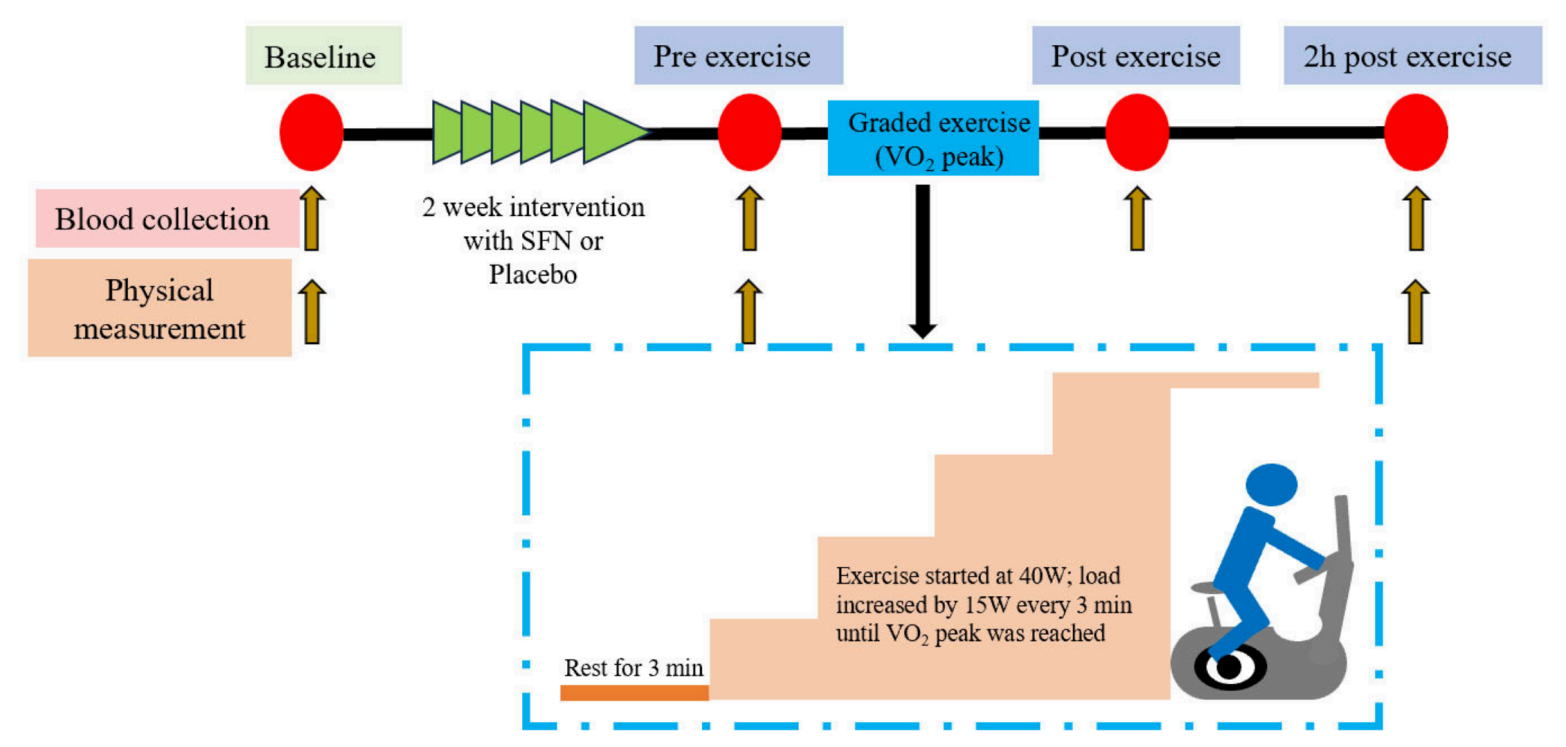
Illustration of the experimental protocol of the study.

The maximal exercise test was performed to determine the peak oxygen uptake (VO_2_ peak) using a cycle ergometer (Powermax-VII, Combi Wellness, Tokyo, Japan) in a temperature-controlled room (temperature 22 °C, relative humidity 50%). The initial power output was set to 40 W (same for both male and female), which was subsequently increased by 15 W every 3 min until either exhaustion or the subject was unable to maintain a cadence of 60 rpm (Figure 1). During the exercise test, the expired gas was continuously collected and calculated every 30 s using an automated gas analyzer system (AE-310S, Minato Medical Science, Tokyo, Japan) and the breath-by-breath method. The gas analyzer was calibrated according to the manufacturer’s instructions before each test. During the last 30 s of each stage, rating of perceived exertion (RPE) was recorded. The data were calculated by averaging the last minute of 3 min stage. The VO_2_ peak (mL·kg^−1^·min^−1^) was recorded as the highest oxygen uptake (VO_2_) value attained during the exercise test. The VO_2_ peak was defined as the VO_2_ average during the last 30 s before exhaustion. Heart rate (HR) was measured using a HR monitor and application (POLAR H10 and Polar Beat, Polar Electro Japan, Tokyo, Japan). The VO_2_ peak was considered achieved if at least two or more of the following criteria were met: (1) VO_2_ plateau, (2) HR reaching 90% of the predicted maximum HR (HRmax) by age (predicted HRmax = 220 − age) [28], and (3) RPE using a Borg scale (Borg 1982) [29] reaching 18 or greater. Subjective evaluation of muscle soreness was evaluated by a visual analogue scale (VAS) at three time points, before exercise (pre), immediately (post) and 2 h after exercise.

### 2.5. Blood Sampling

Blood samples were collected once before supplementation (baseline, prior to the intervention) as the baseline and, after two weeks of supplementation, on the exercise test day at three time points: pre-exercise (prior to exercise but after the intervention with supplements), immediately post-exercise, and 2 h post-exercise. Venous blood samples were collected in ethylenediaminetetraacetic acid (EDTA)-containing tubes or serum separation tubes. The EDTA-containing tubes for plasma separation were immediately centrifuged at 3500 rpm for 13 min at 4 °C. The serum separation tubes were left at room temperature for 30 min and then centrifuged at 3500 rpm for 13 min at 4 °C. All samples were stored at −80 °C until analysis. The total white blood cells (WBCs); red blood cells (RBCs); hemoglobin; hematocrit; and the number of lymphocytes, neutrophils, and monocytes in the blood were measured in the EDTA-treated whole-blood samples using an automatic blood cell counter (pocH100i, Sysmex; Kobe, Japan).

### 2.6. Measurement of Biochemical, Oxidative Stress and Antioxidant Markers

Serum AST, ALT, CK and Mb were measured by BML, Inc. (Tokyo, Japan). Free radical analyzer (Wismerll Co., Ltd., Tokyo, Japan) was used to measure d-ROMs, BAP and OXY levels in serum. Serum biomarkers were adjusted according to percent change in plasma volume (ΔPV) calculated from value of hemoglobin and hematocrit [30].

### 2.7. Statistical Analysis

The sample size was calculated using the program G*power 3.1 [31] to detect an effect size of *f* = 0.5 for the within–between interaction (capsule × time), with a power of 0.8 and a significance level of α = 0.05 under the assumption of a correlation coefficient among repeated measures of *r* = 0.7 and a nonsphericity correction of ε = 0.5. A total of 14 participants were recruited, and all completed the study. The other statistical analysis was performed with statistical software (IBM SPSS version 25, IBM Corp., Armonk, NY, USA). All data are presented as the mean ± standard error (SE). A Shapiro–Wilk test was performed to assess the normality of the data distribution, and the non-normally distributed variables were log-transformed prior to analysis. A Greenhouse–Geisser correction was used to adjust degrees of freedom when the assumption of sphericity was violated. Comparison of subjective components and exercise output were performed using a Student’s *t*-tests. To identify the main effect of capsule (SFN or placebo), a two-way analysis of variance (ANOVA) with repeated measures was performed. Also, a three-way ANOVA was performed to identify the effect of gender, capsule (SFN or placebo) and timeline (pre-exercise, post-exercise and 2 h post-exercise). When significant time effects (pre, post and 2 h) were evident, Tukey’s post hoc test was used to identify the significant differences among the mean values. Statistical significance was defined as *p* < 0.05.

## 3. Results

### 3.1. Subject Characteristics, Physiological Variables and Exercise Output

A total of fourteen healthy adults (age, 25.07 ± 1.04 years; weight, 65.28 ± 4.13 kg; height, 170.07 ± 3.46 cm; BMI, 22.48 ± 0.91) participated in this study and were assigned to the initial supplementation sequence using an alternating allocation procedure. The changes in body composition between the SFN and placebo groups are presented in Table 1. During the maximal exercise test (Table 2), no significant differences were observed in the VO_2_ peak (mL·kg^−1^·min^−1^), exercise time (min), or maximum respiratory exchange ratio (RER) between the SFN and placebo groups. The changes in VAS (ΔVAS) and changes in RPE (ΔRPE) due to exercise showed no significant differences between groups. During the study period no adverse effects were observed or self-reported.

### 3.2. Changes in Blood Cells

**Figure 2:**
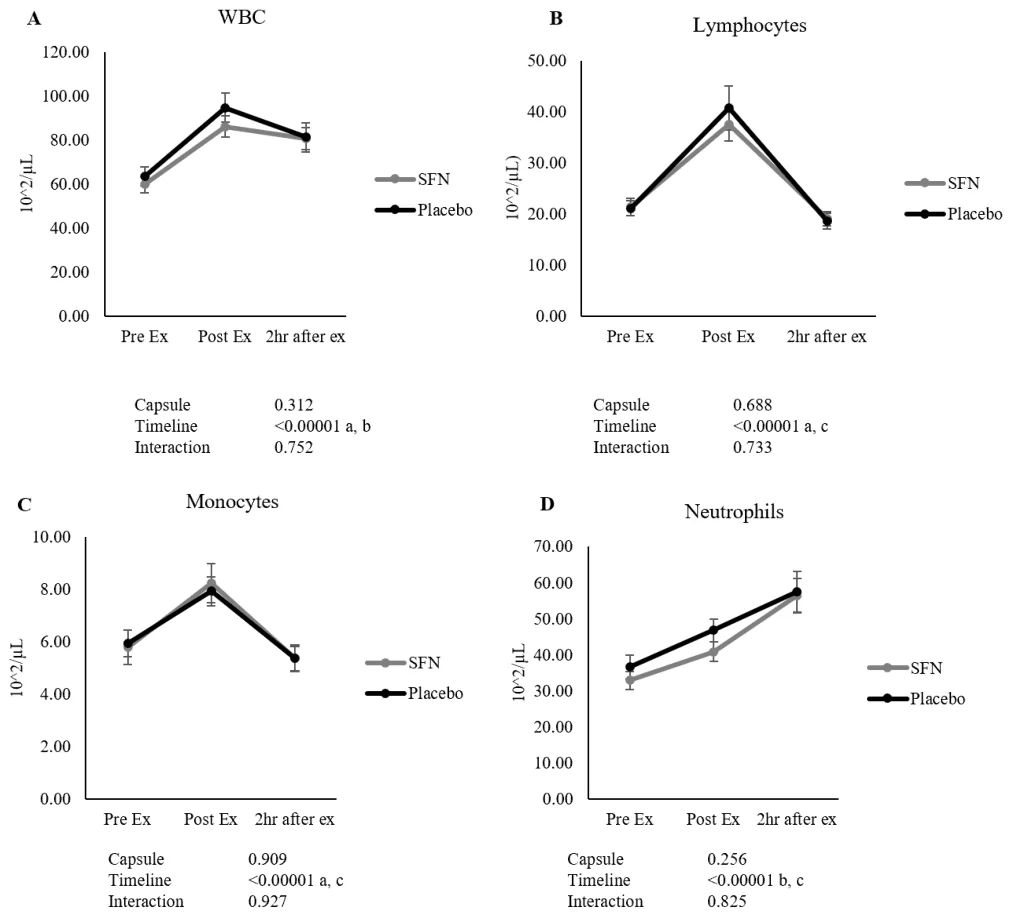
Changes in the number of white blood cells (WBCs) (**A**), lymphocytes (**B**), monocytes (**C**), and neutrophils (**D**). Data are presented as mean ± SE. a, statistical differences between pre-exercise and post-exercise; b, statistical differences between pre-exercise and 2 h post-exercise; c, statistical differences between post-exercise and 2 h post-exercise.

Exercise significantly increased the counts of total white blood cells (WBCs), lymphocytes, monocytes and neutrophils in both groups (Figure 2A–D). The WBCs, lymphocytes and monocytes increased immediately after exercise and gradually returned towards the normal level over time. In contrast, the neutrophil counts increased progressively over time, and peaked at 2 h post-exercise. However, no significant interactions were observed, suggesting that SFN supplementation did not alter the circulating blood cell counts.

### 3.3. Organ and Muscular Injury Markers

As shown in Table 3, the AST and ALT showed no significant differences among the groups or any time points. A significant main effect of capsule was observed for CK (F (1,104) = 4.75; *p* = 0.032; ηp^2^ = 0.044) and Mb (F (1,104) = 7.41; *p* = 0.008; ηp^2^ = 0.067), with lower values observed during the SFN supplementation compared with placebo.

### 3.4. Oxidative Stress and Antioxidant Level Markers

In this experiment, levels of d-ROMs and BAP tests did not show any significant main effect between the capsules. Both in the BAP and OXY tests, the changes were attributable to the exercise itself, irrespective of supplementation. However, when evaluating the changes relative to the baseline, a significant capsule interaction was observed among the variables d-ROMs and OXY. In Table 4, compared to the placebo group, the SFN group showed significantly smaller increases in their d-ROMs levels and a more favorable preservation of OXY levels (*p* < 0.05) following exercise. No significant group differences were found for the percent changes in BAP (*p* = 0.838). These results indicate that SFN may modulate the acute oxidative response to exercise, while supporting endogenous antioxidant defenses.

## 4. Discussion

This double-blind, crossover study evaluated the effects of two weeks of oral sulforaphane (SFN) supplementation on the endurance capacity, oxidative stress, and muscle damage in healthy adults. Short-term SFN supplementation was associated with selective modulation of reactive oxygen metabolites and muscle damage markers, whereas no significant improvement in endurance capacity was observed. The human evidence regarding the effects of SFN supplementation on endurance capacity together with exercise-induced oxidative stress and muscle damage remains limited. While animal studies have demonstrated that SFN enhances endurance capacity by reducing oxidative stress-induced muscle damage during exercise [32], comparable evidence in humans is still limited. The present study therefore extends the available human evidence by simultaneously evaluating endurance capacity, oxidative stress, and muscle damage following short-term SFN supplementation. The exercise protocol used here comprised an incremental cycling exercise test to volitional exhaustion, which has been broadly accepted as a powerful way to provoke oxidative stress and physiological strain among exercise-trained people. Maximal or near-maximal exercise tests have been found in the past to elevate oxidative stress biomarkers like superoxide dismutase (SOD), catalase (CAT), and malondialdehyde (MDA) considerably, even when conducted for relatively short periods of exercise [33]. This lends credibility to the use of our protocol as a good method to measure systemic redox responses when used as a tool to study antioxidant interventions like SFN.

Endurance exercise markedly induces mobilization of circulating WBCs, including leukocytes, lymphocytes, and neutrophils, immediately after exercise. During the recovery phase, lymphocyte counts typically decline below basal values, whereas neutrophil counts remain elevated for several hours [34]. A previous ex vivo study reported that SFN reduces reactive oxygen species (ROS) production in both whole blood and neutrophils in a concentration-dependent manner [11]. In our study, the neutrophil counts increased in both groups following exercise, and the SFN group demonstrated a relatively lower increase than the placebo group. However, the differences were not significant enough for comparison.

In this study, the CK and Mb levels were significantly lower in the SFN group compared with the placebo. For myoglobin, the estimated marginal means were 17.81 (95% CI: 13.35–22.27) for the SFN treatment and 26.47 (95% CI: 22.01–30.94) for the placebo treatment. However, the treatment-by-time interactions were not significant, indicating that the temporal response to exercise was not significantly modified. Both AST and ALT are enzymes typically related to liver function, and are also present in muscle cells. Depending on the intensity and duration of exercise, they are released into circulation along with intramuscular proteins, such as CK and Mb [35,36]. In contrast to the CK and Mb levels, the changes in AST and ALT were limited to within 2 h post-exercise, suggesting that these enzymes may require a longer time to peak following muscle stress. This aligns with previous findings by Pal et al., where significant increases in AST and ALT were observed at 24 and 48 h post-exercise rather than immediately after exercise [37]. Chronic supplementation (four weeks) with SFN suppressed a single bout of high-intensity resistance exercise-induced muscle damage [38]. Previously published articles reported that SFN acts indirectly in the modulation of the muscle redox environment, leading to the prevention of acute exhaustive exercise-induced muscle damage [39,40]. In particular, increased CK activity was a typical marker of muscle damage in many studies and remained increasing until 2–4 days following exercise [12,41,42]. Komine et al. reported that CK activity peaked at 4 days following eccentric exercise; notably, the SFN supplementation group showed a trend toward lower CK values at 2 days post-exercise compared with the control [43]. In addition, leakage of Mb from muscle cells due to exercise may reduce muscle oxygen stores and the metabolic capacity of muscle cells [44]. These findings suggest a potential role of SFN in reducing markers of muscle damage following exercise. However, further studies are needed to determine its clinical relevance and investigate its potential effects on recovery and performance outcomes.

SFN has gained considerable attention owing to its unique health benefits, primarily through the sustained upregulation of antioxidant and detoxification genes, offering a more adaptive and robust response to oxidative stress. In our study, the influence of SFN supplementation on oxidative stress blood biomarkers (d-ROMs, BAP, and OXY tests) was assessed to measure the antioxidant potential of SFN on exercise-induced oxidative stress. Here, we noticed that exercise increased the d-ROMs, BAP, and OXY levels in both groups, but the interaction was not significant enough for comparison. However, the SFN group showed a relatively lower increase in d-ROMs at the post-exercise measurement, which was significant when compared to the percentage change from baseline. In contrast, the OXY levels were relatively elevated in the SFN group compared to those in the placebo group, and a significant main effect of capsule was observed when the percent change was calculated. In addition, the levels of antioxidant biomarkers (BAP) were elevated after two hours of exercise, although the differences were not significant enough to compare. The observed percent changes in the OXY and d-ROMs levels in the SFN group suggests that SFN may contribute to preserving antioxidant capacity and limiting the relative increase in oxidative stress following exercise. Moreover, our findings are in line with a previously published article, where SFN showed both a direct and indirect effect on antioxidant capacity by suppressing neutrophil-derived ROS that exhibit strong antioxidant activity, either as an exogenous ROS scavenger or as an endogenous activator of the Nrf2 signaling pathway [11]. Following endurance exercise, CK and Mb levels typically increase, indicating muscle injury, oxidative stress and inflammation, which subsequently normalizes over time [34,45,46]. The discrepancy between CK and Mb levels may be attributed to their distinct release kinetics following exercise. The molecular mass of Mb (17 kDa) is lower than that of CK (82 kDa), which causes Mb to be released quickly and rapidly cleared from circulation [34]. These exploratory associations suggest a potential relationship between antioxidant capacity and circulating muscle damage markers; however, the small sample size precludes causal inference. Although statistically significant differences were observed for selected biomarkers, the magnitude of these changes and their physiological significance remain uncertain. Notably, no improvement in endurance capacity was detected, suggesting that the observed biochemical changes may represent modest alterations in redox status rather than functionally meaningful improvements in exercise performance. Future studies should determine whether these biomarker changes translate into measurable physiological or performance benefits.

Our findings corroborate those of Flockhart et al. (2023), who demonstrated that daily intake of glucosinolate-rich broccoli sprouts enhanced Nrf2-mediated antioxidant defense and attenuated oxidative stress during high-intensity training [47]. While their study relied on muscle biopsy data and submaximal lactate responses, we extend this evidence by showing that short-term SFN supplementation reduces serum markers of muscle damage (CK, Mb) and modulates oxidative stress responses (d-ROMs, OXY) following maximal exercise. The d-ROMs assay provides a sensitive measure of circulating hydroperoxides and has been shown to correlate with other established oxidative stress biomarkers in both clinical and exercise settings [48]. In addition, d-ROMs levels have been shown to correlate with known oxidative stress biomarkers, including F2-isoprostanes, as well as with electron spin resonance (ESR), the reference method for free radical detection [49,50]. Therefore, while future studies should incorporate additional molecular markers for greater specificity, the use of d-ROMs and BAP in the present study provides a practical and scientifically sound approach for assessing acute oxidative stress responses in humans [48]. Collectively, these findings suggest that the effects of antioxidant supplementation may vary according to the intervention protocol, participant characteristics, exercise modality, and outcome measures evaluated [18,22,32].

Although both male and female participants were included, the study was designed and powered to detect overall treatment effects in a crossover framework rather than sex-specific interactions. The relatively small sample size should also be considered when interpreting the present findings. Although the crossover design reduced between-subject variability, the modest sample size may have limited the statistical power to detect smaller treatment effects and increased the possibility of Type II errors. Larger, adequately powered crossover trials are warranted to confirm these findings. Moreover, while experimental studies have demonstrated that SFN activates the Nrf2 pathway, molecular markers of Nrf2 signaling were not evaluated in the present study. Therefore, this mechanism should be considered a plausible biological explanation rather than as there being direct evidence for it from the current investigation. Although we observed significant main effects of SFN supplementation, the lack of capsule × time interaction limits a definitive attribution of the exercise-induced changes to SFN intake. The participants were not restricted from consuming cruciferous vegetables during the intervention. As these foods contain SFN and related isothiocyanates, their background intake may have introduced inter-individual variability in redox responses. Nevertheless, the crossover design likely minimized systematic differences between conditions. Moreover, blood sampling was performed only up to two hours after exercise, which allowed us to capture acute responses but not longer-term adaptations; future studies should extend the follow-up period to address this gap. However, because the study used a double-blind, crossover design, any such effects would likely have been balanced between conditions. The percentage change analyses presented in this study were intended to provide a descriptive assessment of the relative changes from baseline. Future studies should include stricter dietary controls to minimize this potential source of variability. Despite these limitations, our findings contribute to the preliminary human evidence supporting the redox-modulatory potential of SFN and highlight the need for further mechanistic and longitudinal studies to clarify its role in exercise-induced oxidative stress and recovery.

## 5. Conclusions

The beneficial effects of SFN on health are diverse but not completely elucidated. Additionally, the research on SFN intake is too limited to recommend it as a regular supplement for physically active individuals. These findings provide preliminary evidence that supplementation with a glucoraphanin-containing preparation designed to support SFN formation may influence selected responses to maximal exercise. Nevertheless, the limited sample size, indirect biomarker approach, and absence of functional benefit necessitate cautious interpretation. Larger studies incorporating molecular endpoints and extended follow-up are needed to define the physiological relevance of SFN to exercise-related redox regulation.

## Figures and Tables

**Table 1:** Comparison of body composition analysis.

Variables	Condition	Baseline			Pre-Exercise			2 h Post-Exercise			*p* Value (G, C, T)
		Total	Male	Female	Total	Male	Female	Total	Male	Female	
Height (cm)	SFN/Placebo	170.07 ± 3.46	178.29 ± 1.27	161.86 ± 1.37	-	-	-	-	-	-	<0.0001
Weight (kg)	SFN	65.28 ± 4.13	73.43 ± 3.07	57.13 ± 2.32	65.34 ± 4.17	73.54 ± 3.06	57.13 ± 2.42	65.33 ± 4.16	73.57 ± 3.07	57.09 ± 2.33	<0.0001, NS, NS
	Placebo	65.49 ± 4.35	74.46 ± 3.06	56.53 ± 2.21	65.24 ± 4.15	73.60 ± 3.03	56.87 ± 2.20	65.61 ± 4.14	73.77 ± 3.00	57.44 ± 2.44	
BMI (kg/m^2^)	SFN	22.48 ± 0.91	23.16 ± 1.01	21.80 ± 0.79	22.54 ± 0.91	23.14 ± 1.01	21.93 ± 0.81	22.47 ± 0.93	23.16 ± 1.02	21.79 ± 0.82	<0.01, NS, NS
	Placebo	22.49 ± 0.95	23.47 ± 1.03	21.51 ± 0.77	22.41 ± 0.92	23.20 ± 1.02	21.63 ± 0.76	22.55 ± 0.93	23.27 ± 1.01	21.83 ± 0.83	
SMM (kg)	SFN	28.54 ± 2.68	34.99 ± 1.00	22.10 ± 0.85	28.72 ± 2.65	35.11 ± 1.00	22.33 ± 0.80	28.26 ± 2.69	34.77 ± 0.96	21.76 ± 0.79	<0.0001, NS, NS
	Placebo	28.66 ± 2.79	35.46 ± 0.91	21.87 ± 0.77	28.76 ± 2.66	35.13 ± 1.02	22.39 ± 0.86	28.37 ± 2.69	34.81 ± 1.05	21.93 ± 0.84	
BFM (kg)	SFN	14.11 ± 1.82	11.97 ± 1.86	16.24 ± 1.49	13.81 ± 1.94	11.64 ± 1.98	15.99 ± 1.62	14.56 ± 1.95	12.33 ± 1.96	16.79 ± 1.66	<0.0001, NS, NS
	Placebo	13.94 ± 1.78	12.07 ± 1.92	15.81 ± 1.41	13.61 ± 1.76	11.86 ± 1.98	15.37 ± 1.34	14.54 ± 1.80	12.51 ± 1.69	16.57 ± 1.68	
Percent body fat (%)	SFN	22.08 ± 2.95	15.91 ± 1.91	28.24 ± 1.60	21.55 ± 3.03	15.43 ± 2.06	27.67 ± 1.76	22.73 ± 3.12	16.36 ± 2.04	29.10 ± 1.83	<0.0001, NS, NS
	Placebo	21.70 ± 2.93	15.79 ± 2.01	27.61 ± 1.69	21.29 ± 2.81	15.69 ± 2.10	26.90 ± 1.51	22.66 ± 2.90	16.67 ± 1.71	28.64 ± 1.83	

Data are presented as mean ± standard error (SE). BMI = body mass index; SMM = skeletal muscle mass; BFM = body fat mass; G = gender effect; C = condition effect (SFN or placebo); T = timeline (baseline, pre-exercise, 2 h post-exercise); NS = non-significant.

**Table 2:** Maximal exercise test output.

	SFN			Placebo			*p* Value (G, C)
	Total	Male	Female	Total	Male	Female	
VO_2_ peak (mL·kg^−1^·min^−1^)	29.12 ± 2.16	33.42 ± 2.36	24.82 ± 2.90	30.12 ± 2.17	33.92 ± 2.36	26.32 ± 3.16	0.011, NS
Exercise time (min)	29.57 ± 3.01	37.29 ± 2.69	21.86 ± 3.51	29.57 ± 3.36	38.14 ± 2.51	21.00 ± 4.24	<0.0001, NS
RER	1.07 ± 0.01	1.07 ± 0.02	1.08 ± 0.03	1.08 ± 0.02	1.06 ± 0.02	1.09 ± 0.03	NS, NS
ΔVAS (exercise effect)	5.63 ± 1.77	3.68 ± 0.76	7.58 ± 3.43	4.85 ± 0.63	5.21 ± 1.12	4.49 ± 0.66	NS, NS
ΔRPE	1.75 ± 0.05	1.80 ± 0.07	1.71 ± 0.14	1.65 ± 0.05	1.67 ± 0.05	1.62 ± 0.1	NS, NS

Data are presented as mean ± SE. VO_2_ = volume of oxygen uptake; RER = respiratory exchange ratio; Δ = changes; VAS = visual analogue scale; RPE = rating of perceived exertion; G = gender effect; C = condition effect (SFN or placebo); NS = non-significant.

**Table 3:** Effects on organ and muscle damage markers.

Variables	Normal Range	Pre-Exercise	Post-Exercise	2 h Post-Exercise	Capsule; Time; Interaction
AST (U/L)	11–35				
SFN		21.07 ± 1.57	20.29 ± 1.44	22.36 ± 1.66	0.330; 0.738; 0.978
Placebo		22.64 ± 2.57	22.52 ± 2.49	23.71 ± 2.77	
ALT (U/L)	6–39				
SFN		19.21 ± 4.29	18.14 ± 3.93	19.57 ± 4.44	0.251; 0.940; 0.983
Placebo		15.5 ± 1.87	15.63 ± 2.49	16.5 ± 1.95	
CK (U/L)	M 62–287 F 45–163				
SFN		125 ± 18	121.52 ± 16.89	129.07 ± 17	0.034 *; 0.989; 0.997
Placebo		289 ± 185.05	291.84 ± 133.07	309.28 ± 141.2	
Mb (ng/mL)	<60				
SFN		16.79 ± 1.78	15.82 ± 1.95	22.82 ± 3.66	0.011 *; 0.668; 0.715
Placebo		28.62 ± 6.61	27.31 ± 5.71	28.14 ± 4.83	

Data are presented as mean ± SE. M, male; F, female. * *p* < 0.05.

**Table 4:** Effects on reactive oxygen metabolites (d-ROMs) and antioxidant activity (BAP and OXY-adsorbent tests).

Variables	Normal Range	Pre-Exercise	Post-Exercise	2 h Post-Exercise	Capsule; Time; Interaction
d-ROMs (U.CARR)	250–300				
SFN		295.64 ± 17.02	319.64 ± 20.32	294.07 ± 18.21	0.781; 0.192; 0.923
Placebo		291.79 ± 16.36	325.57 ± 18.81	304.77 ± 17.33	
BAP (µmole/L)	>2200				
SFN		2322.57 ± 33.73	2638.79 ± 43.56	2355.86 ± 25.90	0.407; <0.0001 ^a,b^; 0.114
Placebo		2253.79 ± 31.45	2703.93 ± 49.43	2286.46 ± 36.85	
OXY (µmole/mL)	>350				
SFN		404.07 ± 13.82	441.07 ± 13.47	402.93 ± 9.26	0.969; 0.008 ^a,b^; 0.899
Placebo		409.43 ± 10.08	435.64 ± 10.54	405.07 ± 13.23	
Percent changes in the variables from baseline					
d-ROMs (%)					
SFN		99.81 ± 2.27	107.42 ± 2.39	98.82 ± 1.61	0.038 *; <0.0001 ^a,b^; 0.545
Placebo		100.70 ± 1.42	112.32 ± 2.11	103.49 ± 1.83	
BAP (%)					
SFN		101.45 ± 1.84	115.40 ± 2.74	102.91 ± 1.62	0.838; <0.0001 ^a,b^; 0.323
Placebo		99.15 ± 1.90	119.09 ± 3.10	100.31 ± 2.61	
OXY (%)					
SFN		106.03 ± 3.59	115.97 ± 4.15	105.98 ± 3.08	0.032 *; 0.026 *^,b^; 0.892
Placebo		101.22 ± 3.53	107.73 ± 3.88	99.79 ± 3.49	

Data are presented as mean ± SE. * *p* < 0.05; a, significant differences between pre-exercise vs. post-exercise; b, post-exercise vs. 2 h post-exercise.

## Data Availability

Data are published within this article.
